# Photoacoustic Imaging as a Tool for Assessing Hair Follicular Organization

**DOI:** 10.3390/s20205848

**Published:** 2020-10-16

**Authors:** Ali Hariri, Colman Moore, Yash Mantri, Jesse V. Jokerst

**Affiliations:** 1 Nanoengineering Department, University of California-San Diego, La Jolla, CA 92093, USA; a1hariri@ucsd.edu (A.H.); cam081@eng.ucsd.edu (C.M.); 2 Bioengineering Department, University of California-San Diego, La Jolla, CA 92093, USA; ymantri@eng.ucsd.edu; 3 Material Science and Engineering Program, University of California-San Diego, La Jolla, CA 92093, USA; 4 Radiology Department, University of California-San Diego, La Jolla, CA 92093, USA

**Keywords:** LED-based photoacoustic imaging, hair follicles, FUE, FUT

## Abstract

Follicular unit extraction (FUE) and follicular unit transplantation (FUT) account for 99% of hair transplant procedures. In both cases, it is important for clinicians to characterize follicle density for treatment planning and evaluation. The existing gold-standard is photographic examination. However, this approach is insensitive to subdermal hair and cannot identify follicle orientation. Here, we introduce a fast and non-invasive imaging technique to measure follicle density and angles across regions of varying density. We first showed that hair is a significant source of photoacoustic signal. We then selected regions of low, medium, and high follicle density and showed that photoacoustic imaging can measure the density of follicles even when they are not visible by eye. We performed handheld imaging by sweeping the transducer across the imaging area to generate 3D images via maximum intensity projection. Background signal from the dermis was removed using a skin tracing method. Measurement of follicle density using photoacoustic imaging was highly correlated with photographic determination (R^2^ = 0.96). Finally, we measured subdermal follicular angles—a key parameter influencing transection rates in FUE.

## 1. Introduction

Follicular unit extraction (FUE) and follicular unit transplantation (FUT) are the gold standard surgical interventions for androgenic alopecia and account for >99% of transplant procedures [1,2]. Both of these techniques involve transplantation of healthy hair follicles from a safe donor area to a low-density region of thinning/balding [3,4]. The procedures differ in how the donor follicles are collected. In FUE, follicles are individually extracted using a handheld punching device. In FUT, a strip of scalp is resected and then dissected to obtain individual follicles [5,6]. Consequently, FUE results in less severe scarring than FUT, and is more commonly requested by patients for this reason [7]. However, FUE is a more technically demanding procedure and has a higher risk of follicle transection; in some cases, it can also lead to cyst formation or a “moth-eaten” appearance of the donor region [8].

One important element for both of these procedures is characterization of the follicular units both at the donor and implant site (pre- and post-operatively). Factors such as graft size, angle, and grafting density have a significant effect on follicular unit survival rates [9,10]. Beyond implant survival, these metrics also have implications for potential complications such as the development of subdermal cysts or telogen effluvium (“shock loss”) [11,12]. Finally, variations in graft density and angle can have major impacts on cosmetic satisfaction.

Photography and/or visual inspection is the current gold-standard means of characterization during treatment planning and evaluation of results [1]. However, this practice neglects the subdermal orientation/depth of follicles, which is the primary factor governing undesirable transection rates in FUE [13]. Furthermore, these optics-based inspections cannot evaluate the follicle below the skin surface or during the “shock loss” period after transplant [14]. Indeed, subdermal characterization of the follicles at the donor and implant sites could inform the clinician on the best donor sites as well as the risk and status of downstream complications such as telogen effluvium and cysts. Therefore, we propose the use of a non-invasive imaging modality for gleaning both supra- and subdermal information about the density and orientation of follicles.

Ultrasound is a widely deployed imaging modality across medical specialties with applications in dermatology for melanoma, inflammatory diseases, and lipoablation [15]. These applications are currently being expanded as photoacoustic imaging (PAI)—an augmented form of ultrasound—continues to gain clinical traction [16,17]. PAI uses pulsed light to generate ultrasound waves from the imaging target. This shifts the mechanism of imaging contrast from differences in acoustic refraction to optical absorption. When performed simultaneously with ultrasound (routine practice for commercially available systems), the modality is capable of real-time imaging with anatomical and molecular contrast through many centimeters of tissue. PAI has experienced tremendous research growth in the past decade as a diagnostic platform and is currently being investigated for a number of dermatological conditions [18,19,20,21,22,23,24]. Recently, a laser-based PAI system was proposed for volumetric imaging of a single follicular unit [25]. In this work, we introduce an LED-based photoacoustic imaging method for fast and non-invasive characterization of follicle density and subdermal angle with an emphasis on its diagnostic value in FUE and FUT.

## 2. Materials and Methods

### 2.1. Photoacoustic Imaging System

In this work, we used a AcousticX CYBEDYNE LED-based photoacoustic imaging system from CYBERDYNE Inc. (formerly Prexion) (Tokyo, Japan) [26]. The system is equipped with a 128-element linear array ultrasound transducer with a central frequency of 10 MHz and a bandwidth of 80.9% fitted with two LED arrays. The imaging equipment could be used with a variety of wavelengths. We used both 690 and 850 nm LED arrays in this study. We found that 690 nm provides more information about the skin layer and 850 nm shows more penetration depth.

The repetition rate of these LEDs is tunable between 1, 2, 3, and 4 KHz. The pulse width can be changed from 50 to 150 ns with a 5-ns step size. The transducer can be scanned to generate three-dimensional (3D) data using a maximum intensity projection (MIP) algorithm. The lateral and axial resolution of this imaging system is ~550 and 260 µm, respectively. This system can detect blood vessels to a depth of ~1.5 cm [26].

### 2.2. Imaging and Data Collection

The study enrolled a single adult healthy Caucasian male; the subject provided written informed consent. All work was conducted with approval from the UCSD Institutional Review Board and was in accordance with the ethical guidelines for human subject research set forth by the Helsinki Declaration of 1975. In order to maintain a sterile imaging environment, we used sterile CIV-Flex probe cover (CIVCO Medical Solution, Coraville, IA, USA) on the photoacoustic transducer.

The images were acquired using acoustically transparent coupling gel (clear image singles ultrasound scanning gel, Next medical product company, Somerville, NJ, USA), and we evaluated several regions of the body with varying follicle density. First, we imaged the nape of the subject’s neck to include both skin and hair in the same field of view. We then imaged the parietal region of the scalp after trimming the hair with clippers (Wahl Clipper Corporation, Sterling, IL, USA). We used both ultrasound and photoacoustic mode to image the follicle density on the scalp.

In order to show that this technique can image hairs that are not visible (subdermal hairs), we imaged the abdomen at baseline, trimmed to leave a residual ~1 mm of hair (Wahl trimmer), and shaved with a razor (Gillette Mach 3, Boston, MA, USA). To further evaluate the capacity of the imaging technique to evaluate follicle density, we also imaged the subject’s arm (little hair; no trimming or shaving) and face (freshly shaved). These various regions of the body were also photographed while protecting the subject’s anonymity.

The imaging experiments used both 690 and 850 nm excitation wavelengths. The scanner was swept by hand at ~1 cm/s across the skin collecting both ultrasound and PA data for subsequent 3D visualization via maximum intensity projection (MIP)—a volume rendering that projects the voxels with maximum intensity in the correspondence plane [27,28]. Photographs were also taken at each imaging site for comparison of hair density.

### 2.3. Data Analysis

All imaging data were recorded as rf data and reconstructed using Fourier transform analysis (FTA) [29]. Images were exported and analyzed as bmp files types. The ImageJ 1.48 v toolbox was used to analyze all the data [30]. We used both B-mode cross-sections [15,31] and MIP volumes [23] to evaluate the capabilities of our LED-based photoacoustic imaging system for this application. In all images, ultrasound pixel intensities are shown in 8-bit grayscale, and photoacoustic pixel intensities are shown as hot color maps. In order to measure follicle densities, we compared the region of interest (ROI) for a photoacoustic MIP image to a photograph (2 × 2 cm^2^ for both). To identify the follicles, we combined two metrics: photoacoustic intensity and morphology of the image. We evaluated the background photoacoustic intensity histogram, and we found that the background had a mean 8-bit pixel intensity lower than 35. Therefore, if intensity values are higher than 35, we set a threshold to define those data to be a feature—i.e., vein or follicle. Next, we could visually distinguish between vein and follicle structures. Each photoacoustic image and photograph were divided into four quadrants, and the number of follicles was counted manually. Subdermal follicular angles were measured with respect to the dermis using the angle tool function in ImageJ.

### 2.4. Statistical Analysis

Follicle densities for each region were quantified by averaging the counts for each quadrant of the ROI, and error bars represent the standard deviation across these four areas. We used a Student’s t-test to assess statistical differences between the measurement techniques.

## 3. Results

**Figure 1:**
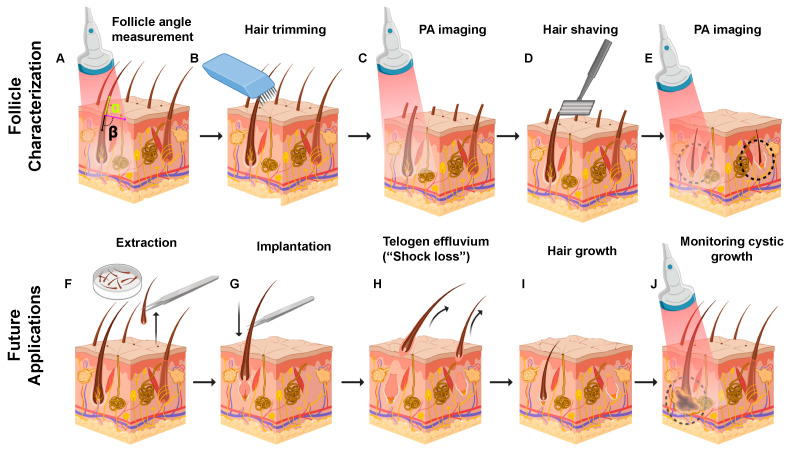
Photoacoustic imaging of follicular organization. The upper panel shows current work. (**A**) The follicular angle under the skin can be determined using photoacoustic imaging (PAI). (**B**) Hair trimming, (**D**) shaving, and subsequent (**C**,**E**) PA imaging to detect individual hair follicles, subdermal roots, and follicular angles. Future applications in follicular unit extraction and transplantation (FUE and FUT). (**F**) Hair follicles are excised from a safe donor area. (**G**) Extracted follicles are implanted in the donor spot. (**H**) Original hair shaft sheds while the dermal papilla is retained in a process called telogen effluvium (“shock loss”). PAI can quickly confirm successful implantation by imaging hair growth within the papilla before the follicle is visible on the skin surface. (**I**) Hair growth from successfully transplanted follicles. (**J**) Follicles implanted too deep can result in cyst formations 1–2 years after the procedure. PA imaging can monitor this cystic growth.

The main goal of this study was to evaluate photoacoustic ultrasound as a diagnostic and staging tool for hair transplant procedures including FUE. Figure 1 shows the current and potential clinical workflow for the implementation of photoacoustic imaging to evaluate follicular organization. Figure 1A illustrates the value of photoacoustic imaging in pre-surgical staging in FUE: the images could be used to measure subdermal follicular angles (alpha and beta). Since these angles affect transection rate and can lead to more hair loss [32], a threshold angle could be established such that follicles below this value are not chosen for extraction. Follicle characterization steps are detailed in Figure 1B–E. These steps demonstrate that hair trimming and shaving followed by photoacoustic imaging can detect individual hair follicles, subdermal roots, and follicular angles. Future applications in follicular unit extraction and transplantation (FUE and FUT) are detailed in Figure 1F–J highlighting the value of imaging at the short and long-term post-implantation stages. Specifically, the implanted follicles can be visualized prior to eruption from the dermis immediately post-implantation (Figure 1G), during telogen effluvium to verify the presence of the residual papilla (Figure 1E), or periodically long-term (over 12–24 months) to monitor the formation of cysts from improper implantation (Figure 1J).

### 3.1. Hair Follicle Imaging Using PAI

**Figure 2:**
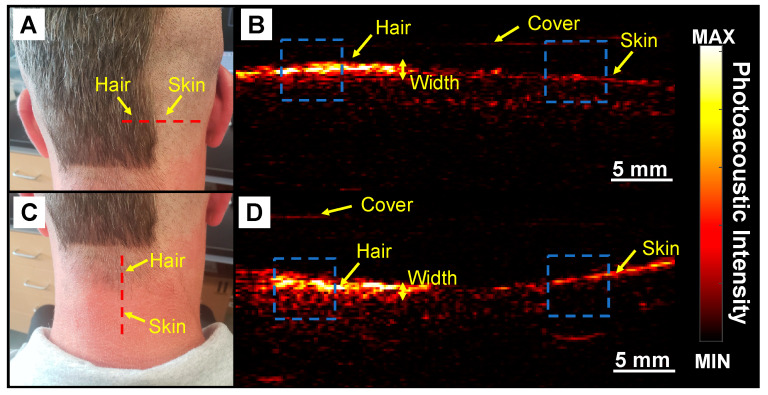
Hair imaging using PAI. This experiment contains positive and negative controls to validate the imaging technique using 690 nm light source. (**A**) Photograph of unshaved and shaved portions of the scalp. Red dashed line represents the imaging plane. (**B**) Photoacoustic image of dashed line in A which includes the skin and full hair. Photoacoustic intensity of hair in full hair area is 4.23-fold higher than shaved skin. (**C**) Photograph of the nape with lower hair density than the scalp. Red dashed line represents the imaging plane in D. (**D**) Photoacoustic image along red dashed line in C. Photoacoustic intensity in this area was 2.8-fold higher than the surrounding skin. Blue dashed squares are the region of interest (ROI) used to measure the PA intensity on the skin and hair.

**Figure 3:**
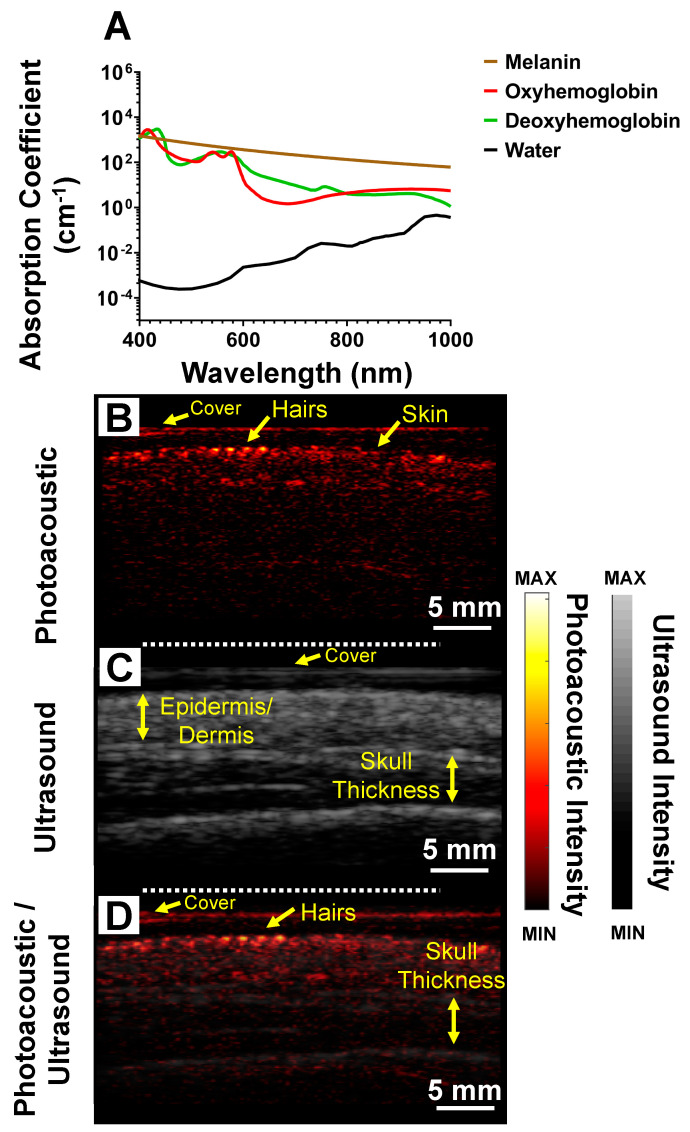
Trimmed scalp imaging. (**A**) Absorption spectra of melanin, oxyhemoglobin, deoxyhemoglobin, and water. Data from http://omlc.ogi.edu/spectra/. (**B**) Photoacoustic image of trimmed scalp. Individual hair follicles are represented by discrete spots on the scalp. (**C**) Ultrasound image from same plane of A including measurements of skull thickness from the ultrasound data. The skull thickness for this subject was 6.05 ± 0.64 mm. (**D**) Photoacoustic and ultrasound overlay allows us to locate hair follicles with respect to the anatomical features of the skull.

**Figure 4:**
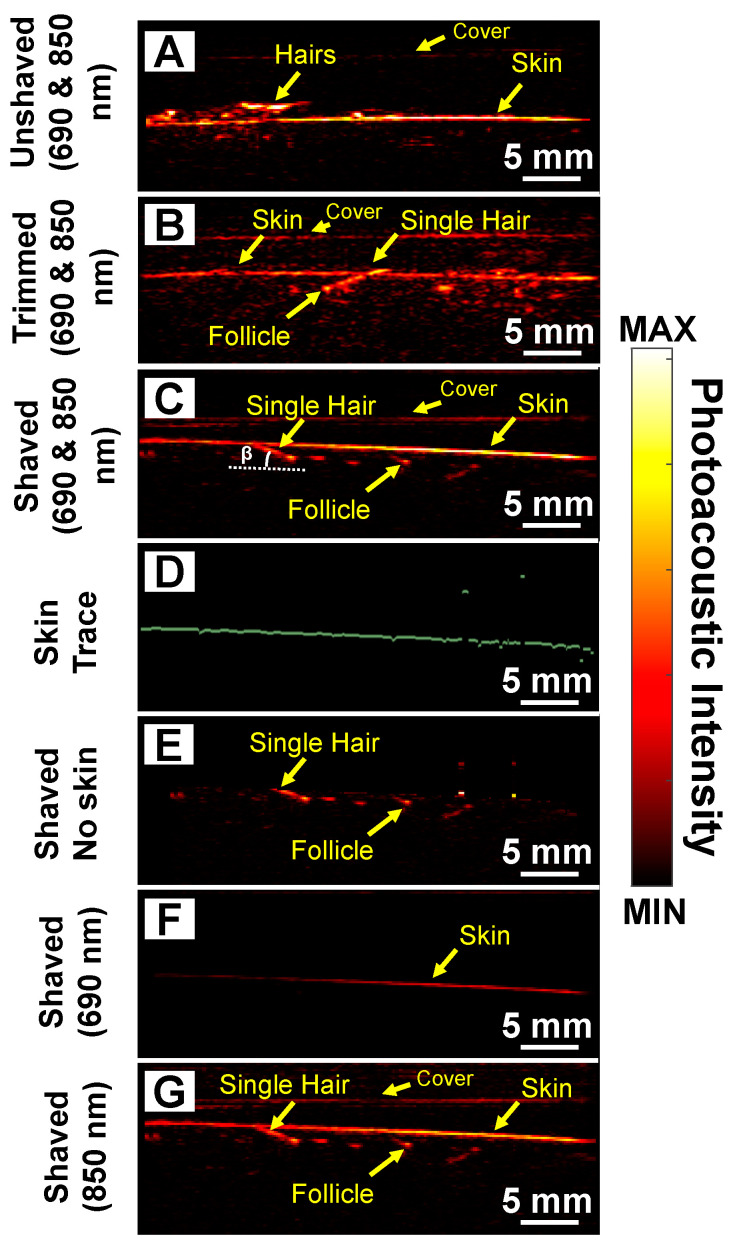
Subdermal imaging. Photoacoustic image of (**A**) unshaved and untrimmed, (**B**) trimmed, and (**C**) shaved abdominal skin using both wavelengths (690 and 850 nm). We could detect individual roots and subdermal hair strands using PAI after trimming and shaving. (**D**) We used a skin tracer algorithm to remove the photoacoustic contribution from the skin. (**E**) Photoacoustic image of shaved area after removing the photoacoustic signal of the skin via digital image processing. Photoacoustic image of the shaved region using (**F**) 690 and (**G**) 850 nm. Melanin in the skin absorbs strongly at 690 nm limiting depth penetration. Imaging at 850 nm allows deeper penetration allowing visualization of both the roots and subdermal strands.

**Figure 5:**
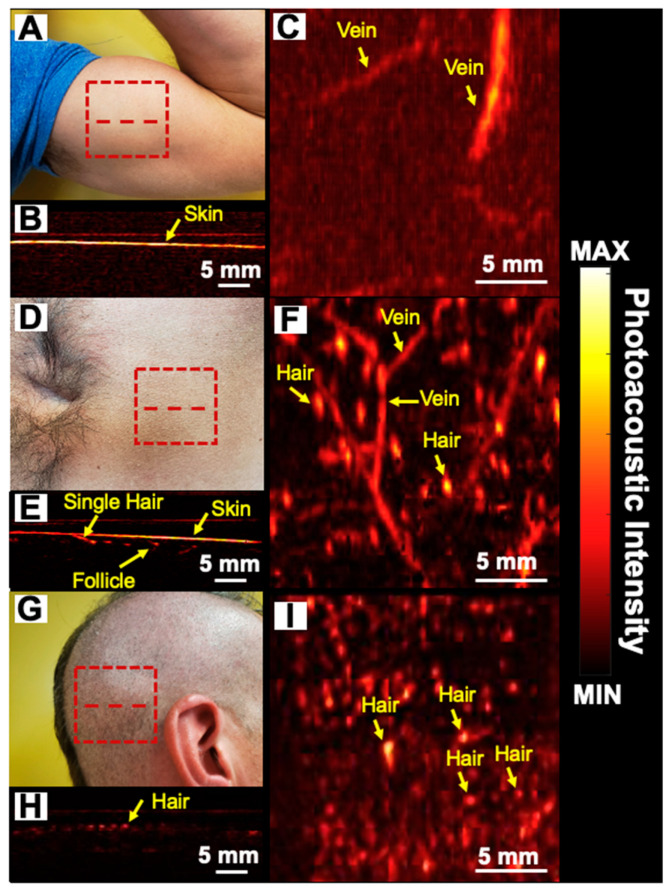
Follicular density images. (**A**,**D**,**G**) photograph; (**B**,**E**,**H**) cross-sectional photoacoustic image along the red dashed line. (**C**,**F**,**I**) maximum intensity projection (MIP) photoacoustic images of biceps, shaved stomach, and trimmed scalp using 850 nm LED light source within the 2 × 2 cm^2^ area marked by red dashed rectangles, respectively. PAI shows the absence of hair on the biceps whereas subdermal hair follicles can be seen in the stomach and scalp regions. Vasculature close to the skin surface can also be imaged due to hemoglobin that absorbs at near-infrared wavelengths and produces photoacoustic signal.

**Figure 6:**
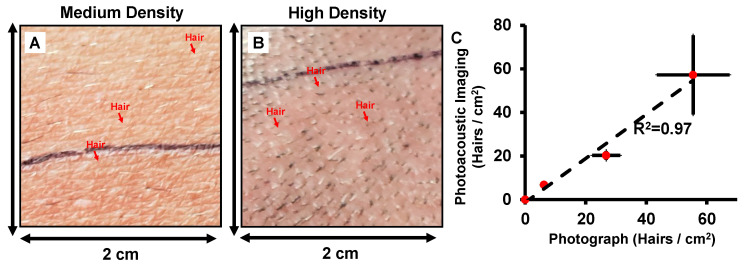
Quantification of follicle density. (**A**,**B**) Photograph of 2 × 2 cm^2^ of medium-density (abdomen) and high-density (scalp) areas. (**C**) Correlation between follicle density measurement using photoacoustic data and photograph images. High correlation (R^2^ = 0.97) is observed between these two techniques.

We first used 690 nm LED light source and did some positive (hair area) and negative (skin area) control experiments to confirm that hair produced PA signal (Figure 2). We then used PAI to image below the skin, measure follicle angle, and determine follicle density (Figure 3, Figure 4, Figure 5 and Figure 6). Figure 2A shows a photograph of the nape of the neck with unshaved and shaved hair. The corresponding PA image is presented in Figure 2B taken along the dashed line in Figure 2A. The PA intensity of unshaved hair in this area was 4.23-fold higher than shaved scalp. These data show that the PA signal corresponds to hair volume. Our subject had gray-brown hair color. Ford et al. demonstrated that gray and blond color generate significantly lower photoacoustic signal than black hair due to the higher melanin content in black hair [25].

We further confirmed this approach by imaging an area with varying hair density. The nape of the neck has decreasing hair density along a plane moving down the spine (Figure 2C). This subject also had recent sun exposure and with red skin in areas not covered by hair. Figure 2D shows a photoacoustic image collected in the red dashed line in Figure 2C. We note the low signal for the bare skin relative to the area with hair. The freshly shaved skin has a lower signal than the nape of the neck because the nape had been exposed to sun and had more pigmentation (Figure 2D). The intensity of hair on the nape was 2.8-fold higher than the surrounding skin. We used the photoacoustic intensity line profile of the hair and measured the average full width at half maximum (FWHM) for the width of the PA profile in Figure 2B,D to be 1.02 ± 0.19 and 0.37 ± 0.09 mm, respectively.

This shows that the width of PA signal can also be an acceptable metric to quantify the hair density. Scanning a 2 × 2 cm^2^ area took 10 s to collect the raw data, 15 s to reconstruct the data, and up to 5 min to process. Future work can integrate automated image-processing tools to streamline the workflow [33]. It is also important to mention that, since the width of transducer is small (4 cm), we are easily able to perform multiple scans from multiple directions and surfaces such as flat and curved.

Figure 3A shows the absorption spectra of melanin, oxyhemoglobin, deoxyhemoglobin, and water. All data were downloaded from http://omlc.ogi.edu/spectra/. For hemoglobin, we used molar extinction coefficient (e) from [34], and the absorption coefficient was measured using the following: $\mu_{a}=\frac{\left(2.303\right)e\left(150\frac{gHb}{liter}\right)}{66,500\;gHb/mole}$. For melanin’s $\mu_{a}$ correspondence to skin, we used $\mu_{a}=1.70\times \;10^{12}\;\lambda^{-3.48}$, where, λ is the wavelength [35]. Using the same imaging setup, we performed PAI and ultrasound on the trimmed (with no guard) region of the scalp (see Figure 5G for exact region imaged). Figure 3B–D show the photoacoustic, ultrasound, and overlay of photoacoustic and ultrasound, respectively. With PAI, individual hair follicles are seen as discrete spots on the scalp Figure 3B. Ultrasound imaging was used to measure skin thickness of 3.78 ± 0.28 mm which is within reported values of 2–6 mm for normal adult humans [36]. The skull thickness was measured to be 6.05 ± 0.64 mm which is consistent with literature values ~6.5 mm for adult men [37,38].

### 3.2. Subdermal Imaging

Next, we evaluated the utility of this technique for hair that was not visible as a model for the subdermal papilla that might be present after shock loss during transplant (Figure 1E) and used the abdominal region as a model. Figure 4A shows a photoacoustic image (690 and 850 nm as wavelength) of unshaved stomach with multiple hair follicles present above the skin surface. We next removed an increasing amount of hair and first trimmed the region for a residual ~2 mm of hair present on the skin surface (Figure 4B). While the photoacoustic signal present on the skin surface decreases, the signal from the root remains. We next shaved the skin to remove all hair protruding from the skin surface while leaving the subdermal region intact. Figure 4C shows that PAI can image and detect hair follicles and their roots under the skin surface.

Previous studies show that skin surface generates strong photoacoustic signal [39]. This is because it is the first region to encounter the optical pulse and thus has the highest fluence. Skin with high amounts of melanin also has strong photoacoustic signal because melanin absorbs infrared wavelengths of light [40,41] (Figure 3A). Regardless of the source, the strong signal from skin can make detecting follicles under the skin surface challenging. Thus, we used a skin tracing technique (digital image processing) to remove signal from the skin surface to facilitate improved image formation [42,43]. Figure 4D shows the skin trace from Figure 4C. We removed the photoacoustic contribution from the skin surface to obtain Figure 4E. Figure 4F,G show the shaved stomach imaged at 690 and 850 nm, respectively. We observed that most of the 690 nm light is absorbed by melanin in the skin; hence, we cannot image the follicle under it. However, with 850 nm illumination, light is able to penetrate deeper allowing us to image the root and the follicle. The literature shows that absorption for melanin at 690 nm is ~2.07 times higher than 850 nm [44] (Figure 3A).

Some individual hair strands can be seen germinating from the roots, whereas others show discrete spots with no strands. This is expected because not all follicles are perfectly aligned to the plane of the transducer. Our transducer can cover a 3.5-cm field-of-view. The field-of-view in linear array transducers is a function of the number and the distance between the elements (pitch size). Additionally, the pitch size is ~$\frac{1}{2f_{c}}$, where $f_{c}$ is defined as the center frequency of the ultrasound transducer [45]. Therefore, the field of view is a function of the center frequency and number of elements. Higher center frequencies with a constant number of elements will have a smaller field-of-view.

### 3.3. Follicle Density

Follicular density (number of follicles/cm^2^) is important in scouting for donor areas and assessing transplant success [8]. We evaluated the ability of the PAI technique to quantitate follicle density in various regions. For humans, the follicular density varies widely in different areas. For example, the scalp has a higher follicular density than the biceps. The average follicular units and hair density per square centimeter is 65–85 and 124–200, respectively [10]. We identified three areas with varying hair density: the biceps, (Figure 5A), shaved stomach (Figure 5D), and trimmed scalp (Figure 5G) as low, medium, and high-density areas, respectively. We imaged a 2 × 2 cm^2^ area represented by the red dashed rectangle in Figure 5A–C. Figure 5B,E,H show the cross sectional photoacoustic image along the red dashed line from the biceps, shaved stomach, and trimmed scalp, respectively. Figure 5C,F,I represent the MIP photoacoustic map of biceps, shaved stomach, and trimmed scalp, respectively. These MIP maps show both hair and blood vessels. Indeed, the superficial vasculature can also be detected using photoacoustic imaging due to the light absorption from hemoglobin [46]. For PA follicle density evaluation, we used the 850 nm LED light source (longer wavelength, higher penetration depth). These maps are similar to previous studies that showed vascularity using PAI [47,48,49].

Figure 6 further processes this raw data in Figure 5 and quantifies the follicular density along with the fourth data point from the face/beard of the same subject (photographs not included in Figure 5 for anonymity). We plotted the follicles/cm^2^ for both photographs and photoacoustic MIP maps for the four regions and show that the follicular densities change with skin region (Figure 6C). There was good correlation (R^2^ = 0.97) between follicular density measured using PAI and visual counting from photographs. These results show that PAI can quantify hair follicle density across different regions—including in regions where the hair is not visible above the skin surface.

### 3.4. Follicle Angle

The transection rate in FUE can impact outcomes, and one key variable here is the follicle angle: The rate of transection increases when the angles point toward the nuchal lines [32]. We utilized PAI to measure the angle of multiple follicles (*n* = 25) relative to the skin surface on the shaved stomach. The follicular angle (β) was measured to be 18.62 ± 5.281° in this area (Figure 4C). To the best of our knowledge, no other study has reported the follicle angle on the stomach; values reported on the scalp vary from 10° to 40° depending on the location on the scalp [50,51,52].

## 4. Discussion

There are several error sources unique to photoacoustic imaging in this application. First, hair and skin color are key parameters here. Lighter hair and lighter skin color can generate less photoacoustic signal relative to darker skin. Darker skin has higher melanin content, absorbing more light and reducing depth penetration. Second, scanning is performed manually using a hand-held transducer, and the risk of shaking and instability can vary between operators but could be minimized with training. Image processing algorithms can also resolve and remove these artifacts [53,54]. Third, the resolution of our photoacoustic system could limit scanning of very fine hair. The resolution of our imaging system depends on the center frequency of the ultrasound transducer. As we mentioned before, our LED-based imaging system has an axial resolution of 260 µm. If the hair width is too small (lower than resolution range), then our imaging system may not able to resolve it. Similarly, if two follicles are separated by less than ~550 µm (lateral resolution) we cannot resolve both follicles. We could overcome these limitations by increasing the center frequency of the ultrasound transducer.

Previously, Ford et al. demonstrated the structural and functional analysis of hair follicles using volumetric multispectral optoacoustic tomography [25]. That work offered higher resolution images than this work, but we believe that our LED-based photoacoustic imaging system has important advantages in terms of a ~7-fold larger field of view and the use of LED light sources that are more compact, safe, cheap, and rugged.

In future work, we will image follicular features such as density and subdermal angle with the LED-based photoacoustic imaging technique and validate their prognostic value for in vivo models of FUE and FUT. We will also evaluate the technique in patients with a range of skin tones to better understand and study the limitations of the technique. We will also develop an image-processing algorithm to segment the photoacoustic images and measure the follicle density in a more automatic fashion.

## 5. Conclusions

In this study, we evaluated the application of photoacoustic imaging for the fast and non-invasive characterization of follicular density and subdermal angles with an emphasis on its diagnostic value for FUE and FUT. We showed that a portable, inexpensive, and low fluence LED-based imaging system has potential value for these procedures.
